# Psychometric properties of the persian version of the nursing clinical reasoning scale

**DOI:** 10.1002/nop2.2041

**Published:** 2023-11-29

**Authors:** Touba Hosseinzadeh, Nastaran Mirfarhadi, Moluk Pouralizadeh, Kian Norouzi Tabrizi, Masoud Fallahi‐Khoshknab, Hamid reza Khankeh, Forozan Shokooh

**Affiliations:** ^1^ Department of Nursing, School of Nursing and Midwifery Guilan University of Medical Sciences Rasht Iran; ^2^ Department of Nursing, Social Determinants of Health Research Center University of Social Welfare and Rehabilitation Sciences Tehran Iran; ^3^ Department of Nursing University of Social Welfare and Rehabilitation Sciences Tehran Iran; ^4^ Department of Health in Disasters & Emergencies University of Social Welfare and Rehabilitation Sciences Tehran Iran; ^5^ Department of Basic Sciences University of Social Welfare and Rehabilitation Sciences Tehran Iran

**Keywords:** clinical reasoning, factor analysis, nurses, psychometrics, statistical analysis

## Abstract

**Aim:**

The aim of this study was to translate the Nursing Clinical Reasoning Scale (NCRS) into Persian and evaluate its psychometric properties.

**Design:**

This study was a methodological and cross‐sectional study.

**Methods:**

This methodological study was conducted in 2020 in a teaching hospital. After obtaining necessary permission from its developers, NCRS was translated into Persian through the method proposed by the World Health Organization. Then, its face, content, and construct validity and reliability were assessed. For construct validity assessment through exploratory and confirmatory factor analyses, 300 nurses (two 150‐nurse samples), who had randomly been selected, completed the instrument. Reliability also assessed through the internal consistency and the stability methods. Data were analysed using the SPSS (v. 20.0) and the AMOS (v. 5.0) software.

**Results:**

The content validity indices of NCRS and its items were 0.97 and more than 0.79, respectively. Exploratory factor analysis revealed an assessment and confirmation factor and an implementation and reflection factor for the scale which together explained 57.30% of the total variance. Confirmatory factor analysis also confirmed this two‐factor structure (*χ* ^2^/*df* = 2.11, NNFI = 0.952, RMSEA = 0.053, CFI = 0.91, GFI = 0.94, IFI = 0.95, and NFI = 0.96). The Cronbach's alpha and the intraclass correlation coefficient values of the scale were 0.96 and 0.94, respectively.

**Public Contribution:**

The Persian NCRS can help nursing policy makers and mentors identify the need for developing nurses' and nursing students' CR skills and implement need‐based educational courses to improve these skills. Moreover, it helps determine whether the educational programmes are effective in improving nurses' CR skills and clinical competence.

## 1 INTRODUCTION

Nurses work in unpredictable and complex conditions and hence need to make prompt and rational decisions in order to provide safe and effective care. Quality decision making relies on advanced thinking skills such as critical thinking, creative thinking, and clinical reasoning (CR) (Brown Tyo & McCurry, 2019). CR is a clinical competency in the heart of nursing practice (Goudreau & Létourneau, 2014; Simmons, 2010) that helps nurses effectively perform their complex roles (Benner, 2012). Clinical reasoning process facilitates the meaningful interpretation of patients' problems and the development of effective care planning (Modi et al., 2015).

CR skills development is facilitated by efficient nursing education, effective nursing management, and professional development of nursing (Hosseinzadeh et al., 2022). Evidence shows that CR skills development has significant relationship with the quality of nursing education, professional expertise of nursing instructors, and quality educational environments (Alfayoumi, 2019; Forsberg et al., 2014; Herron et al., 2016; Hunter & Arthur, 2016; Oostra et al., 2019; Yauri & Nash, 2019). Effective teaching‐learning strategies and innovative educational models are also necessary to develop CR skills (Georg, 2014; Tesoro, 2012; Wuryanto et al., 2017; Yauri & Nash, 2019).

Quality CR has many different positive outcomes. Clinical reasoning enables nurses to interpret patient data to determine patterns and problems, establish accurate diagnoses, and identify the best patient care measures (Gracia‐Lewis, 2013). Contrarily, poor CR is associated with nurses' inability to assess patient and environmental conditions, interpret and synthesize clinical data (Fox & Draycott, 2014), and establish accurate diagnoses, and hence, can endanger patient safety (Lapkin et al., 2010). Nonetheless, nursing education in Iran pays little attention to the development of nurses' CR skills and most nurses informally learn these skills (Santos et al., 2019). Since the nurses might not be able to connect the clinical evidences into a meaningful pattern, their clinical reasoning skills are usually described as poorly organized knowledge structures. Moreover, due to the lack of valid and specific scales in the nursing field, the evaluation of clinical reasoning skills in nursing education in Iran has certain limitations and not measured in official evaluations (Safabakhsh & Jahantigh, 2017).

## 2 BACKGROUND

Evidently, clinical reasoning could be developed through education; therefore, the prerequisite to achieve this level of development would be education and continuous assessment of clinical reasoning skills in nurses. Therefore, the first step to develop nurses' CR is to accurately assess this process using valid and reliable instruments. Previous studies used different instruments for CR assessment. For example, some studies used the Script Concordance Test as an innovative and standard instrument to assess nursing students' CR (Côté & St‐Cyr Tribble, 2012; Dawson et al., 2014 May; Deschênes et al., 2011 jul; Goudreau & Létourneau, 2014). However, it has been emphasized that this form of assessment in nursing is in early stages; therefore, further research is needed to warrant to fully evaluating the reliability and validity of the SCT in assessing of clinical reasoning skills in nursing (Dawson et al., 2014 May). Another instrument for CR assessment is the Clinical Reasoning Evaluation Simulation Tool (CREST) which assesses CR skills in assessing and responding to a clinical satiation. However, CREST does not cover all metacognitive skills, and there are limited reliable data about its validity and reliability (Liaw et al., 2018). Another instrument in this area is the Clinical Reasoning Assessment Rubric (Orrock et al., 2014). This instrument also assesses the fundamental attributes of CR, and there are no data about its construct validity.

The Nursing Clinical Reasoning Scale (NCRS) is another instrument for CR assessment developed by Liou et al. in Taiwan for the self‐assessment of CR skills among hospital nurses and nursing graduates. This instrument has acceptable psychometric properties and can assess and determine the strengths and weaknesses of the CR development process (Liou et al., 2016). NCRS is comprehensive, simple, and congruent with the nursing process and some studies introduced NCRS as a valid and reliable instrument to assess CR skills among nursing students and newly graduated nurses (Holder, 2018; Johnston, 2019). However, this instrument had not yet been translated into Persian and had not culturally been adapted for the context of Iran. Therefore, the present study was conducted to translate NCRS into Persian and evaluate its psychometric properties.

## 3 THE STUDY

### 3.1 Aim

This study aimed to validate the Scale in the Persian language. The objectives of the study were

1. To translate the scale into Persian language.
2. To test the validity [face, content, exploratory factor analysis (EFA) and confirmatory factor analysis (CFA)].
3. To test the reliability [alpha and intraclass correlation coefficient (ICC)] of the Persian version of Nursing Clinical Reasoning Scale.

### 3.2 Method

This methodological study was conducted in 2020. The study had two main phases, namely the translation and cultural adaptation phase and the psychometric evaluation phase.

#### 3.2.1 The translation and cultural adaptation phase

NCRS is a fifteen‐item instrument for CR assessment developed based on the theory of CR (Liou et al., 2016). This theory considers CR as a rational process in which nurses collect and process the data to determine patient problem, develop and use measures to manage patient problem, evaluate the outcomes, and learn patient management. Items are scored from 1 (“Completely disagree”) to 5 (“Completely agree”) and hence, the total score of the scale is 15–75, with higher scores showing greater CR skills. For NCRS translation into Persian, necessary permission was obtained from its developers and then, the scale was translated into Persian through the four‐stage method proposed by the World Health Organization. The four stages of this method are forward translation, expert panel back‐translation, pre‐testing and cognitive interviewing, and development of the final version (WorldHealthOrganization, 2020). Initially, two English‐Persian translators independently translated NCRS from English into Persian. The translators were not only asked to translate the scale but also to provide all possible equivalents for the words and expressions in the main English NCRS. Then, study authors, two instrument development specialists, and two CR experts collectively assessed and compared the two translations and generated a single Persian translation of NCRS. After that, two other translators independently back‐translated the Persian NCRS into English. The same experts assessed and compared their translations and generated a single English translation of NCRS using their translations. The translated English NCRS and the original English NCRS were sent to the developers of the original NCRS who confirmed the conceptual similarity of the two versions of the scale. After that, the study authors and a medical education specialist assessed and confirmed the cross‐cultural equivalence and appropriateness of the Persian NCRS. For face validity assessment, thirty nurses were interviewed in a pilot study to determine any ambiguity or repetition in the items and determine appropriate alternatives for them. The Persian NCRS was revised according to their comments.

#### 3.2.2 The psychometric evaluation phase

In this phase, the face, content, and construct validity as well as the reliability of the Persian NCRS were assessed.

##### Face validity assessment

Face validity was assessed using qualitative and quantitative methods. The qualitative face validity of NCRS was assessed by the same thirty nurses in the pilot study. To this end, the nurses were asked to determine the importance of the scale's items A 5‐point Likert (1 = unimportant; 5 = very important). Quantitative face validity was assessed through calculating item impact score and items with impact score 1.5 or more were considered appropriate (Mohammadbeigi & Aligol, 2015; Petersen et al., 2010). The impact score calculation formula was, $\text{Impact score}=\frac{\sum \left(\text{Frequency}\times \text{Importance}\right)}{N}$

##### Content validity assessment

Content validity was assessed using content validity ratio (CVR) and content validity index (CVI). For CVR calculation, twelve experts in nursing (*n* = 6), medical education (*n* = 4), and instrument development (*n* = 2) were invited to rate item essentiality on a three‐point scale as either “Essential”, “Useful but not essential”, and “Unessential”. They were also asked to comment on item wording and the items were revised according to their comments. Their rating scores were used to calculate CVR based on the Lawshe's method. As the number of experts was twelve, the minimum acceptable CVR value was considered to be 0.56 (Ayre & Scally, 2014). The CVR calculation formula was, $\mathrm{CVR}=\frac{n_{e}-N/2}{N/2}$.

For CVI calculation, the same twelve experts were asked to rate item relevance on a four‐point scale. Then, item CVI was calculated through the following formula, $\mathrm{CVI}=\frac{\text{Number of experts rating the item}\;3\;\text{or}\;4}{\text{Total number of experts}}$. Items with CVI values more than 0.79 were considered acceptable, items with CVI values between 0.70 and 0.79 needed revision, and items with CVI values less than 0.7 were considered unacceptable and were omitted (Polit et al., 2007). Scale‐level CVI (S‐CVI) was also calculated through averaging all item CVI values. An S‐CVI value of 0.9 or more indicates good content validity (Polit & Beck, 2006).

##### Construct validity assessment

Construct validity was assessed through both exploratory and confirmatory factor analyses conducted using the AMOS software. For construct validity assessment, 300 nurses (two 150‐nurse samples), completed the scale. In exploratory factor analysis, a sample of 150 nurses was selected through systematic sampling from a teaching hospital. Systematic sampling was used to select a sample from target population systematically and randomly. All participants were placed on a list. Then, the sampling interval (k) was calculated by dividing the population size by the desired sample size (150 for each factor analysis). According to the preparation of a list of the target population, a starting point was selected at random on the list and from there were selected every kth member of the population to be included in the sample. Inclusion criteria were full‐time employment in the study setting for at least one whole year before the study, an educational level of bachelor's degree or higher, agreement for participation, and no managerial position. Sample size was determined based on a rule of thumb which considered that 5–10 participants per item were adequate (Santos et al., 2016). Participants completed a demographic questionnaire and NCRS. Exploratory factor analysis was performed with principal component method and varimax rotation. The minimum acceptable factor loading value was considered to be 0.3. Sampling adequacy was tested using the Kaiser‐Meyer‐Olkin (KMO) test and a test value more than 0.5 was considered acceptable (Ebadi et al., 2017). The Bartlett's sphericity test was also used to test model appropriateness. Factor number was determined using scree plot and eigenvalues more than 1. For construct validity assessment through confirmatory factor analysis, 150 eligible nurses were randomly selected from the same setting to complete NCRS. Model fit indices were Chi‐square divided by degree of freedom (*χ* ^2^/*df*; acceptable value >3), comparative fit index (CFI; acceptable value <0.9), goodness of fit index (GFI; acceptable value <0.9), normed fit index (NFI; acceptable value ≥0.95), non‐normed fit index (NNFI; acceptable value ≥0.95), and root mean square error of approximation (RMSEA; acceptable value ≤0.05).

##### Reliability assessment

The reliability of NCRS was assessed using the internal consistency and the stability methods. In internal consistency assessment, Cronbach's alpha was calculated for the scale and its factors, and Cronbach's alpha values more than 0.7 were considered acceptable (Polit, 2013). In stability assessment through the test–retest method, 25 nurses from the coronary care unit of the study setting twice completed NCRS with a two‐week interval and then, test–retest intraclass correlation coefficient (ICC) was calculated. ICC values equal to 0.8 and more were considered acceptable (DeVon et al., 2007).

### 3.3 Data analysis

Data were analysed using the SPSS (v. 20.0) and the AMOS (v. 5.0) software. Statistical methods for data analysis were Cronbach's alpha, ICC, and exploratory and confirmatory factor analyses.

### 3.4 Ethical considerations

The Ethics Committee of the. Participants were informed of the study aim and methods, were ensured of confidential data management, and were asked to sign the informed consent form of the study. Completion of NCRS took only 5 min.

## 4 RESULTS

### 4.1 Face validity assessment

One item was revised in qualitative face validity assessment and item impact scores of all items were more than 1.5 in quantitative face validity assessment. Therefore, all fifteen items of NCRS had acceptable face validity.

### 4.2 Content validity assessment

In qualitative content validity assessment, some items were revised respecting their wording and grammar. In quantitative content validity assessment, item CVI values were more than 0.79 and S‐CVI was 0.97 and hence all fifteen items of the scale had acceptable content validity.

### 4.3 Construct validity assessment

A total of 150 nurses participated in construct validity assessment. Most of them were female (90%) and had bachelor's degree (92.66%). The means of their age and work experience were 34.62 ± 4.81 and 8.41 ± 2.017 years, respectively.

**FIGURE 1:**
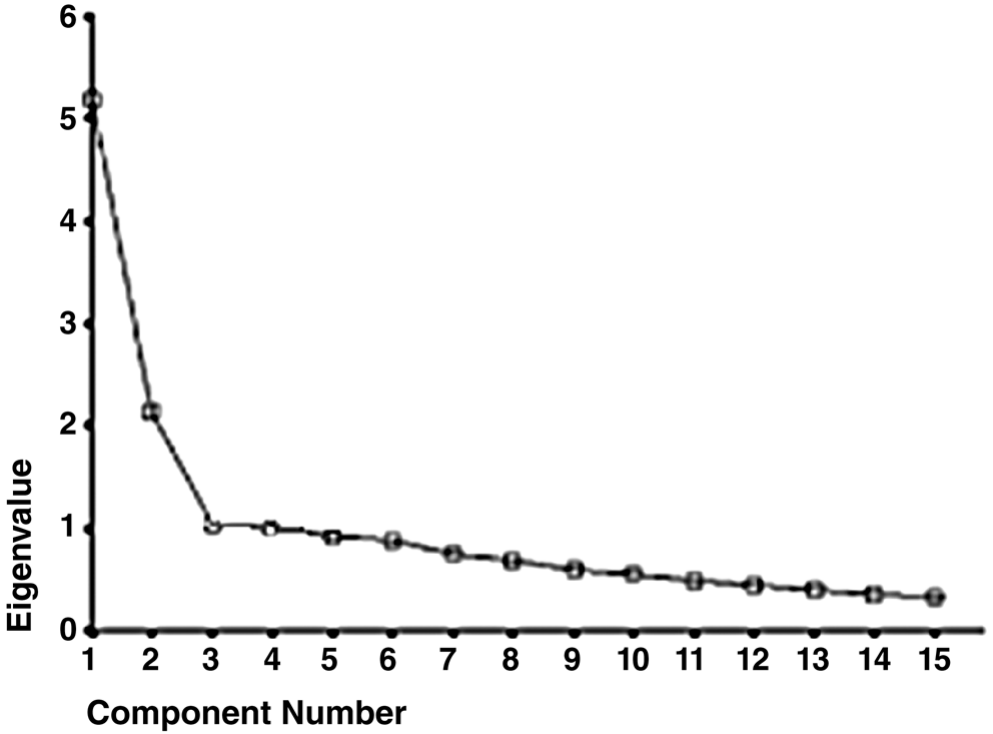
The scree plot to determine the number of the Persian NCRS factors.

**FIGURE 2:**
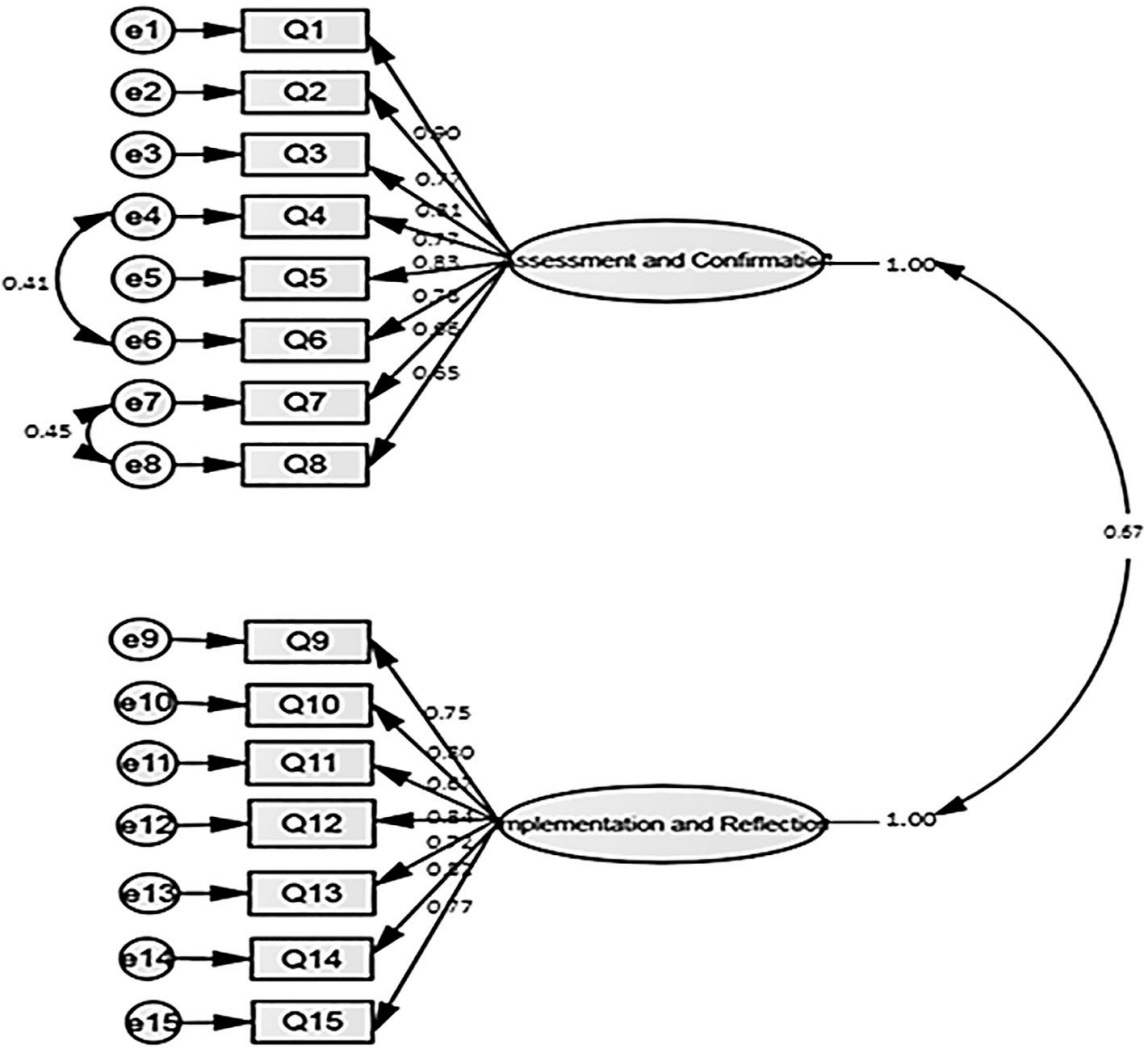
The final factor structure of the Persian NCRS.

The KMO test value was 0.83, and the result of the Bartlett's test value was significant which confirmed sampling adequacy and factor analysis model appropriateness. The scree plot revealed that NCRS consisted of two factors (Figure 1). Exploratory factor analysis with principal component method and varimax rotation also revealed a two‐factor structure for the scale. The two factors of NCRS explained 57.3% of the total variance. Each of the fifteen items of NCRS had a factor loading value of more than 0.3 and was loaded only on one factor (Table 1). Based on their items, the factors were labelled as assessment and confirmation (with eight items) and implementation and reflection (with seven items) (Table 1). Confirmatory factor analysis also confirmed the good fit of the two‐factor model of NCRS (*χ* ^2^ = 83.2267; *p* < 0.001). All goodness of fit indices were also in the acceptable range and confirmed the appropriateness of the two‐factor model (Table 2). Items had strong correlation with their corresponding factors, and there was significant correlation between the measurement errors of items 4 and 6 and items 7 and 8 (Figure 2).

**TABLE 1:** The extracted factors of the Persian NCRS.

Factors	Items	Factor loading	% variance
Assessment and confirmation	I can identify the early signs and symptoms of the deterioration of patient's conditions.	0.751	29.55
	I know how to rapidly collect patient data.	0.750	
	I can accurately apply assessment skills to collect current patient data.	0.711	
	I can identify abnormal cases among the collected patient data.	0.684	
	I can clearly identify critical data based on patient's current conditions.	0.673	
	I can identify patient's health problems based on the collected data.	0.654	
	I can accurately explain the mechanism of the development of patient problems.	0.661	
	I can explain the mechanism and the progress course of signs or symptoms when patients' health condition is getting worse.	0. 633	
Implementation and reflection	I can precisely prioritize and manage each of the identified patient problems.	0.762	27.75
	I can accurately set nursing goals for the identified patient problems.	0.759	
	I can predict medical orders based on the presented patient data.	0.750	
	I can provide appropriate nursing care interventions for the identified patient problems.	0.743	
	I am completely aware of each presented nursing care measure.	0.737	
	I can precisely assess patient status and determine whether the patient has achieved recovery.	0.699	
	I know which follow‐up measures should be perform if there is no improvement in patient conditions.	0.668	

**TABLE 2:** The confirmatory factor analysis model fit indices.

*χ* ^2^	*χ* ^2^/*df*	NNFI	RMSEA (90%CI)	CFI	GFI	IFI	NFI
2267.83	2.11	0.952	0.053 (0.033–0.067)	0.91	0.94	0.957	0.967

### 4.4 Reliability assessment

Internal consistency assessment showed that the Cronbach's alpha and the ICC values of NCRS and its factors were more than 0.9 (Table 3).

**TABLE 3:** The Cronbach's alpha and the intraclass correlation coefficient values of the Persian NCRS.

Factors	Item number	Alpha	ICC
Assessment and confirmation	8	0.95	0.92
Implementation and reflection	7	0.93	0.91
Total	15	0.96	0.94

## 5 DISCUSSION

This study was conducted to translate NCRS into Persian and evaluate its psychometric properties. Findings revealed the acceptable face, content, and construct validity and reliability of the Persian NCRS.

In content validity assessment, the CVI values of NCRS items were 0.95–1, and the S‐CVI of the scale was 0.97. These findings confirm the strong relationship of the NCRS items with the concept of CR (Liou et al., 2016). In agreement with our findings, a previous study confirmed the acceptable content validity of the Italian version of NCRS (Notarnicola et al., 2020).

Construct validity through exploratory factor analysis showed that NCRS has an assessment and confirmation factor and an implementation and reflection factor which together explained 57.30% of the total variance, denoting the appropriateness of the number of factors (Kaplan & West, 2012). The factors of a valid instrument should explain at least 50% of its total variance (Streiner, 1994). Factor analysis in the study of Liou et al., the developers of the original NCRS, showed a single‐factor structure for NCRS which explained 50.7% of the total variance (Liou et al., 2016). A study into the psychometric properties of the Dutch NCRS also showed that the scale had two factors, namely collecting patient data and acting based on collected patient data, which explained 60.4% of the total variance and the factor loading values of the items of these two factors were 0.63–0.8 and 0.56–0.8, respectively (Janssen, 2021). Moreover, KMO test value in the present study was 0.83. KMO test values more than 0.8 indicate the appropriateness of the factor analysis model (Polit et al., 2007).

Confirmatory factor analysis in the present study also confirmed the two‐factor structure of the Persian NCRS. All model fit indices of the NCRS were in the acceptable range. A study into the psychometric properties of the Dutch NCRS also found that the CFI and Tucker‐Lewis index model fit indices were in the acceptable range, while the RMSEA index was not in the acceptable range and hence the model did not fit. Its authors attributed the non‐fit of the model to factors such as sociocultural differences and issues in the translation of the scale into Dutch and noted that considering more than one factor for the scale may improve its mode fit (Janssen, 2021).

We also found significant correlation between the measurement errors of items 4 and 6 and items 7 and 8. Measurement error occurs when items are not accurately identified or are not directly measured. It may also be due to the conceptual similarity of two items (Sharifi et al., 2021). A general look at the semantic structure of items 4 and 6 and items 7 and 8 also shows their conceptual similarity.

The Cronbach's alpha values of NCRS and its factors were more than 0.9, denoting limited conceptual dispersion among NCRS items and great internal consistency and reliability of the scale. Liou et al. also found that the inter‐item correlation and the Cronbach's alpha values of the original NCRS were 0.5 and 0.94, respectively (Liou et al., 2016). Another study also showed the great reliability of the scale with a Cronbach's alpha of 0.90 (Notarnicola et al., 2020). Moreover, the Cronbach's alpha of the Dutch NCRS was 0.94, denoting that the scale is reliable, and its items have strong correlation with each other (Janssen, 2021).

Study findings also showed that the ICC of NCRS was 0.94, which implies the repeatability of the data obtained through it over time. Two previous studies into the psychometric properties of NCRS also reported that its ICC value was 0.85 and 0.90 (Liou et al., 2016; Notarnicola et al., 2020). ICC values more than 0.8 confirm acceptable stability. Therefore, the Persian NCRS is a stable instrument.

## 6 CONCLUSION

The Persian NCRS has acceptable validity and reliability for CR assessment in nursing. The main benefit of NCRS is to determine the level of clinical reasoning skills of nurses, in order to help them to develop effective thinking habits that guide the delivery of high quality, safe, effective, and efficient patient‐centered care.It is expected that using the NCRS will provides scientific data to confirm the necessity of designing educational programs to develop and improve nurses' clinical reasoning competence. In addition, nursing administrators can use the results of evaluating clinical reasoning skills to provide appropriate support in the process of developing professional competence.

## AUTHOR CONTRIBUTIONS

TH: Conceptualization, methodology, investigation, analysis, data curation, writing – reviewing and editing, writing – original draft. NM: Conceptualization, methodology, analysis, data curation. MP: Conceptualization, methodology, analysis, data curation, validation, writing – reviewing and editing. KNT: Supervision, methodology, analysis, data curation, validation. MFK: Supervision, methodology, analysis. HK: Conceptualization, methodology, analysis, data curation. FS: Conceptualization, methodology, analysis, data curation.

## FUNDING INFORMATION

The authors disclosed receipt of the following financial support for the research, authorship, and/or publication of this article: This research was funded by a grant from the University of Social Welfare and Rehabilitation Sciences, Tehran, Iran.

## CONFLICT OF INTEREST STATEMENT

The authors declare no conflict of interest.

## ETHICAL APPROVAL

IR.USWR.REC.1399.073.

## Data Availability

Data available on request due to privacy/ethical restrictions. The data that support the findings of this study are available on request from the corresponding author. The data are not publicly available due to privacy or ethical restrictions.
